# Using Thermodynamics to Predict the Outcomes of Nitrate-Based Oil Reservoir Souring Control Interventions

**DOI:** 10.3389/fmicb.2017.02575

**Published:** 2017-12-19

**Authors:** Jan Dolfing, Casey R. J. Hubert

**Affiliations:** ^1^School of Civil Engineering and Geosciences, Newcastle University, Newcastle upon Tyne, United Kingdom; ^2^Department of Biological Sciences, University of Calgary, Calgary, AB, Canada

**Keywords:** thermodynamics, sulfate reduction, nitrate injection, petroleum reservoir, souring control, biocompetitive exclusion, sulfide oxidation, denitrification

## Abstract

Souring is the undesirable production of hydrogen sulfide (H_2_S) in oil reservoirs by sulfate-reducing bacteria (SRB). Souring is a common problem during secondary oil recovery via water flooding, especially when seawater with its high sulfate concentration is introduced. Nitrate injection into these oil reservoirs can prevent and remediate souring by stimulating nitrate-reducing bacteria (NRB). Two conceptually different mechanisms for NRB-facilitated souring control have been proposed: nitrate-sulfate competition for electron donors (oil-derived organics or H_2_) and nitrate driven sulfide oxidation. Thermodynamics can facilitate predictions about which nitrate-driven mechanism is most likely to occur in different scenarios. From a thermodynamic perspective the question “Which reaction yields more energy, nitrate driven oxidation of sulfide or nitrate driven oxidation of organic compounds?” can be rephrased as: “Is acetate driven sulfate reduction to sulfide exergonic or endergonic?” Our analysis indicates that under conditions encountered in oil fields, sulfate driven oxidation of acetate (or other SRB organic electron donors) is always more favorable than sulfide oxidation to sulfate. That predicts that organotrophic NRB that oxidize acetate would outcompete lithotrophic NRB that oxidize sulfide. However, sulfide oxidation to elemental sulfur is different. At low acetate HS^−^ oxidation is more favorable than acetate oxidation. Incomplete oxidation of sulfide to S^0^ is likely to occur when nitrate levels are low, and is favored by low temperatures; conditions that can be encountered at oil field above-ground facilities where intermediate sulfur compounds like S^0^ may cause corrosion. These findings have implications for reservoir management strategies and for assessing the success and progress of nitrate-based souring control strategies and the attendant risks of corrosion associated with souring and nitrate injection.

## Introduction

**Figure 1:**
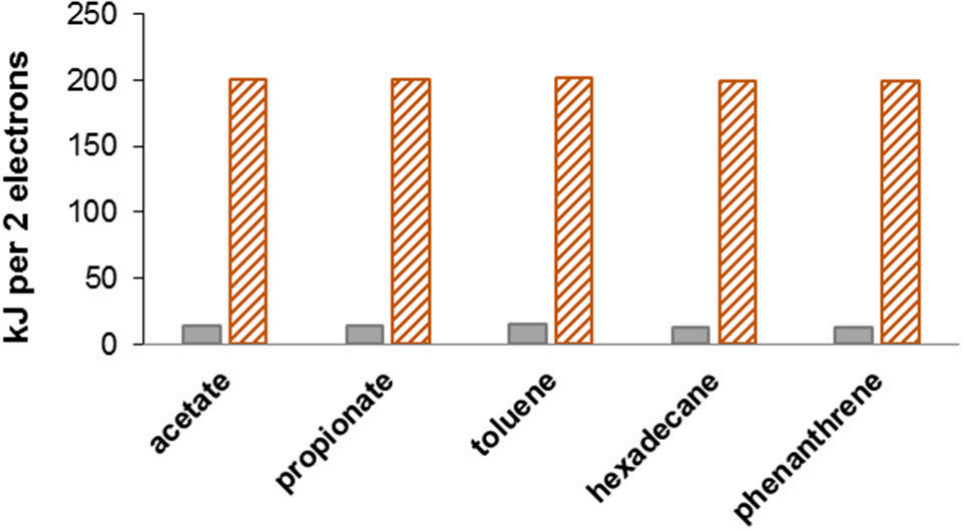
Nitrate (hatched orange bars) is a better electron acceptor than sulfate (gray bars). Energy yield (Δ*G*°′ in kJ/2electrons) is essentially independent of the type of organic electron donor used. In this example nitrate reduction is via denitrification to N_2_ (gas), while sulfate is reduced to sulfide. Partial reactions: acetate oxidation, CH_3_COO^−^ + H^+^ + 2H_2_O → 2CO_2_ + 8[H], Δ*G*°′ = 14.3 kJ/2electrons; propionate oxidation, CH_3_CH_2_COO^−^ + H^+^ + 4H_2_O → 3CO_2_ + 14[H], Δ*G*°′ = 14.2 kJ/2electrons; toluene oxidation, C_6_H_5_CH_3_ + 14H_2_O → 7CO_2_ + 36[H], Δ*G*°′ = 15.4 kJ/2electrons; hexadecane oxidation, C_16_H_34_ + 32H_2_O → 16CO_2_ + 98[H], Δ*G*°′ = 12.9 kJ/2electrons; phenanthrene oxidation, C_14_H_10_ + 28H_2_O → 14CO_2_ + 66[H], Δ*G*°′ = 13.4 kJ/2electrons; denitrification, 2H^+^ + 2${\text{NO}}_{3}^{-}$ + 10[H] → N_2_ + 6H_2_O, Δ*G*°′ = −224.1 kJ/2electrons; sulfate reduction, 2H^+^ + ${\text{SO}}_{4}^{2-}$ + 8[H] → H_2_S + 4H_2_O, Δ*G*°′ = −38.0 kJ/2electrons; $G_{\text{f}}^{{}^{\circ}}$ values for toluene and hexadecane are from Helgeson et al. (1998); $G_{\text{f}}^{{}^{\circ}}$ for phenanthrene is from Dolfing et al. (2009).

**Figure 2:**
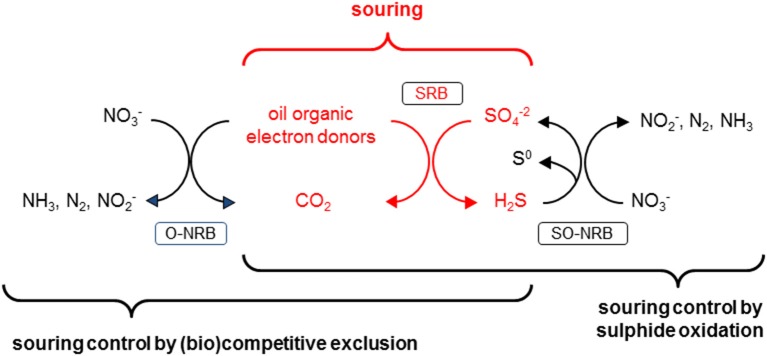
Schematic depicting reservoir souring (microbial production of H_2_S) in oil reservoirs due to the activity of sulfate reducing bacteria (SRB) that use organics present in the oil as electron donors. This souring scenario can interact with two potential mechanisms of nitrate-dependent souring control: (i) biocompetitive exclusion, where organotrophic nitrate-reducing bacteria (NRB) outcompete SRB for common electron donors (i.e., O-NRB), and (ii) sulfide oxidation, where sulfide-oxidizing NRB (i.e., SO-NRB) re-oxidize sulfide produced by SRB to S^0^ and/or ${\text{SO}}_{4}^{2-}$.

Souring, the undesirable production of hydrogen sulfide (H_2_S) in oil reservoirs by sulfate-reducing bacteria (SRB), is a common problem during secondary oil recovery when sea water is injected into the reservoir to maintain the high pressures required for oil extraction (Vigneron et al., 2017). The high concentration of sulfate in seawater (≈ 28 mM) promotes the growth and activity of sulfate reducing bacteria. Introducing sulfate to environments rich in reduced compounds such as hydrocarbons, organic acids and possibly H_2_ (from anaerobic metabolism; Muyzer and Stams, 2008) creates ideal conditions for SRB, hence souring poses ubiquitous challenges for oil producers, especially at offshore operations (Table 1, reactions R1 and R2). Nitrate injection into oil reservoirs can prevent and remediate souring by stimulating the growth and activity of nitrate-reducing bacteria (NRB) (e.g., Telang et al., 1997; Gittel et al., 2009). Two conceptually different mechanisms for NRB-facilitated souring control have been proposed (Hubert et al., 2009; Hubert, 2010). (i) *Nitrate-sulfate competition*: Nitrate is a better (energetically more favorable) electron acceptor than sulfate (Figure 1) (Table 1, e.g., reactions R3–R5 vs. R1, or R7 vs. R2); therefore NRB outcompete SRB, and consequently nitrate injection suppresses sulfate reduction. (ii) *Nitrate driven sulfide oxidation*: The sulfide produced by SRB during souring is re-oxidized with nitrate as electron acceptor (Table 1, R8). The latter mechanism potentially results in a cryptic sulfur cycle (i.e., regenerated sulfate can be re-used by SRB if electron donors are available; Hubert et al., 2003) but as long as nitrate is available sulfide will be essentially absent from the system. As such the two mechanisms are potentially stoichiometrically identical (nitrate-driven oxidation of electron donors either directly or via intermediate S cycling), with the essential difference between them being (Figure 2) that in pathway (i) organotrophic nitrate reducers dominate, while pathway (ii) hinges on the activity of sulfide oxidizing nitrate reducers.

**Table 1:** Stoichiometry and change in Gibbs free energy values for reactions potentially involved in nitrate based souring control.

**Reaction**	**Reactants**	**Products**	**Δ*G*°**	**Δ*G*°′**	**Δ*G*°′**
			**kJ/reaction**		**kJ/2 electrons**
R1	${\text{SO}}_{4}^{2-}$+ 4H_2_ + H^+^	HS^−^+ 4H_2_O	−192.1	−152.1	−38.0
R2	${\text{SO}}_{4}^{2-}$+ CH_3_COO^−^+ 2H^+^	HS^−^+ 2CO_2_ + 2H_2_O	−137.1	−57.2	−14.3
R3	2${\text{NO}}_{3}^{-}$+ 5H_2_ + 2H^+^	N_2_ + 6H_2_O	−1200.6	−1120.7	−224.1
R4	${\text{NO}}_{3}^{-}$+ H_2_	${\text{NO}}_{2}^{-}$+ H_2_O	−158.1	−158.1	−158.1
R5	${\text{NO}}_{3}^{-}$+ 4H_2_ + 2H^+^	${\text{NH}}_{4}^{+}$ + 3H_2_O	−679.7	−599.8	−149.9
R6	CH_3_COO^−^+ H^+^ + 2H_2_O	2CO_2_ + 4H_2_	95.0	55.0	13.8
R7	8${\text{NO}}_{3}^{-}$+ 5CH_3_COO^−^+ 13H^+^	4N_2_ + 10CO_2_ + 14H_2_	−4527.4	−4007.8	−200.4
R8	8${\text{NO}}_{3}^{-}$+ 5HS^−^+ 3H^+^	5${\text{SO}}_{4}^{2-}$+ 4N_2_	−3841.9	−3722.0	−186.1
R9	2${\text{NO}}_{3}^{-}$+ 5HS^−^+ 7H^+^	N_2_ + S^0^ + 6H_2_O	−1261.1	−981.4	−196.3
R10	CH_3_COO^−^+ 4S^0^ + 2H_2_O	2CO_2_ + 4HS^−^+ 3H^+^	103.4	−16.5	−4.1
R11	${\text{NO}}_{3}^{-}$+ 4HS^−^+ 6H^+^	${\text{NH}}_{4}^{+}$ + S^0^ + 3H_2_O	−728.1	−488.5	−122.1
R12	${\text{NO}}_{3}^{-}$+ HS^−^+ H^+^ + H_2_O	${\text{NH}}_{4}^{+}$ + ${\text{SO}}_{4}^{2-}$	−487.6	−447.6	−111.9

Differentiating between causative agents of successful souring control following nitrate application is vital to better understand and further utilize nitrate injection as an emerging technology in the oil and gas sector. Here we use a thermodynamic approach to evaluate which nitrate reduction pathway is most likely to occur in different industrially encountered scenarios. Our considerations include the possibility of nitrate-driven sulfide oxidation to elemental sulfur (Table 1, R9), which has implications for corrosion of oil field infrastructure. The findings can contribute to predictive reservoir souring management strategies as well as assessments of souring- and nitrate-associated microbial-influenced corrosion risks.

## Materials and methods

### Thermodynamic calculations

The amount of free energy available from a reaction depends on the Gibbs free energies of formation of substrates and products as given by the relationship Δ*G*^0^ = Σ$G_{\text{f}}^{{}^{\circ}}$ (products) - Σ$G_{\text{f}}^{{}^{\circ}}$ (reactants). Δ*G*^0^ is the increment in free energy for the reaction under standard conditions: 25°C, 1 atm pressure for gases, 1 molal concentrations for solutes, pH = 0 (also 1 molal; Hanselmann, 1991). For biological systems the conventional standard conditions is as above, but with pH 7 (Thauer et al., 1977). This is considered in Δ*G*°′ values. Δ*G*°′ is identical with Δ*G*° except that the standard conditions of the H^+^ ion is that of pH 7, i.e., $G_{\text{f}}^{{}^{\circ}\prime }$ (H^+^) = −39.95 kJ/mol (Thauer et al., 1977; Dolfing et al., 2010).

Under environmentally relevant conditions the concentrations of reactants and products are generally not identical to those under standard conditions. This is considered in Δ*G*′ values. For a hypothetical reaction *a*A + *b*B → *c*C + *d*D, Δ*G*′ values were calculated by using the mass equation:

$$\begin{array}{l} \Delta G^{\prime }=\Delta {G{}^{\circ}}^{\prime }+\text{RT ln }\left({\left[\text{C}\right]}^{c}.{\left[\text{D}\right]}^{d}/{\left[\text{A}\right]}^{a}.{\left[\text{B}\right]}^{b}\right) \end{array}$$

Gibbs free energy of formation ($G_{\text{f}}^{{}^{\circ}}$) and enthalpy of formation ($H_{\text{f}}^{{}^{\circ}}$) values (used to make temperature corrections for temperatures other than the standard temperature of 25°C) were taken from Hanselmann (1991) with gases (CO_2_ and H_2_) in the gaseous phase, and all other compounds except hexadecane in the aqueous phase. Values for hexadecane were for hexadecane in the liquid state (Helgeson et al., 1998).

Temperature corrections for Δ*G*° were made with the Gibbs-Helmholz equation according to:

$$\begin{array}{l} \Delta G_{\text{Tact}}^{0}=\Delta G_{\text{Tref}}^{0}.\left({\text{T}}_{\text{act}}/{\text{T}}_{\text{ref}}\right)+\Delta H_{\text{Tref}}^{0}.\left({\text{T}}_{\text{ref}}-{\text{T}}_{\text{act}}\right)/{\text{T}}_{\text{ref}} \end{array}$$

with T in K; T_ref_ = 298.15 K (Dolfing et al., 2008; Dolfing, 2015).

## Results and discussion

### The thermodynamic approach

**Figure 3:**
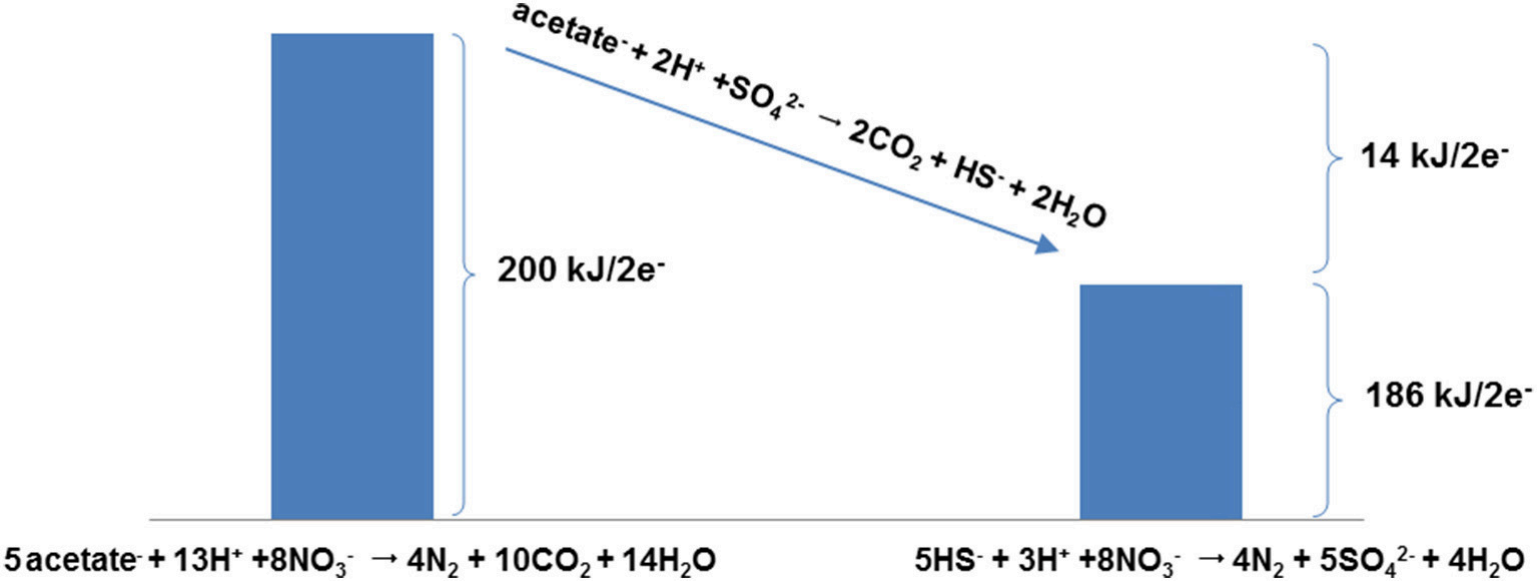
Energetics (Δ*G*°′ per 2 electrons) for the O-NRB reaction 5CH_3_COO^−^ + 13H^+^ + 8${\text{NO}}_{3}^{-}$ → 4N_2_ + 10CO_2_ + 14H_2_O and the SO-NRB reaction 5HS^−^ + 3H^+^ + 8${\text{NO}}_{3}^{-}$ → 4N_2_ + 5${\text{SO}}_{4}^{2-}$ + 4H_2_O, illustrating that the difference in Δ*G*°′ per 2 electrons between the two reactions corresponds to the Δ*G*°′ per 2 electrons for acetate-fuelled souring, i.e., for the reaction CH_3_COO^−^ + 2H^+^ + ${\text{SO}}_{4}^{2-}$ ⇆ 2CO_2_ + HS^−^ + 2H_2_O. This observation allows the acetate-driven sulfate reduction reaction to be used as a predictive tool for assessing whether acetate or sulfide is a better electron donor, i.e., depending on conditions driving the reaction to the left or the right.

From a thermodynamic perspective the question “Which reaction yields more energy, nitrate driven oxidation of sulfide or nitrate driven oxidation of organic compounds (e.g., acetate)?” can be rephrased as: “What is the equilibrium constant for the acetate driven sulfate reduction?” or more to the point: “is acetate driven sulfate reduction exergonic or endergonic?” Figure 3 answers these questions and illustrates this line of reasoning by comparing Δ*G*°′ values for nitrate driven acetate oxidation (Table 1, R7) and nitrate driven sulfide oxidation (Table 1, R8), showing that the former is more exergonic than the latter (200 kJ/2 electrons transferred vs. 186 kJ/2 electrons transferred, under standard conditions). Figure 3 also depicts how the difference between the energetics of these two reactions (14 kJ/2 electrons transferred) represents the energetics of the sulfate driven oxidation of acetate (Table 1, R2). Thus, one can simply consider the energetics of the sulfate driven oxidation of acetate, and whether it is exergonic or endergonic under various conditions, to delineate under which conditions nitrate driven acetate oxidation is more favorable than nitrate driven sulfide oxidation.

**Figure 4:**
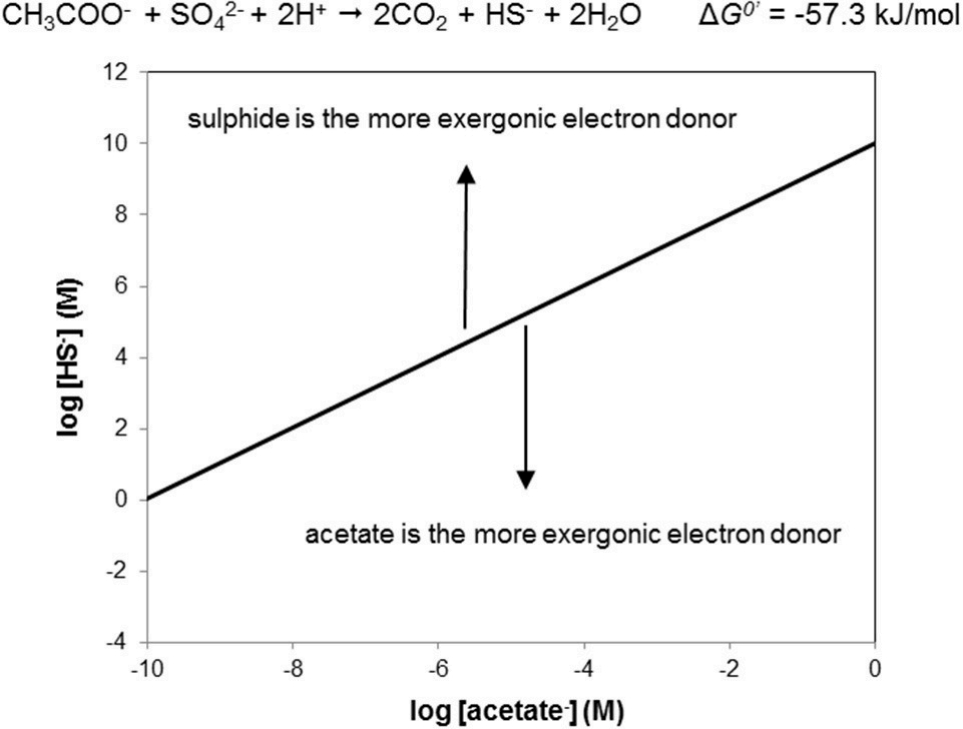
The window of opportunity for acetate driven denitrification (O-NRB) vs. sulfide driven denitrification (SO-NRB), illustrating at which combinations of sulfide and acetate either reaction is most exergonic. Above the black line sulfide is the most exergonic electron donor, below the black line acetate is the most endergonic electron donor. In oil fields the sulfide concentration is never higher than 1 M (34,000 mg L^−1^). Thus, acetate will always be the favored electron donor when nitrate is in large supply and assuming sulfide is completely oxidized to sulfate (as expected when nitrate is in large supply; cf. Figure 8). Calculations are for standard conditions (reactants and products at 1 M aqueous concentrations or at 1 atm partial pressure, at pH 7 and a temperature of 25°C). Reaction stoichiometries are as shown in Figure 3.

Using the sulfate driven oxidation of acetate as a “tool” in this way, we can delineate under which conditions either sulfide or acetate as NRB electron donors are energetically favored. As an example, Figure 4 shows the “window of opportunity” for acetate driven sulfate reduction. The line depicts the combination of HS^−^ and acetate concentrations where the Δ*G* of the reaction is zero, that is, where the reactants and products are in thermodynamic equilibrium (Dolfing et al., 2008). Above the line, the Δ*G* of the reaction is positive, i.e., under these conditions sulfide is the more exergonic electron donor for NRB. Below the line, acetate is the more exergonic electron donor for NRB. The predictive value and conclusion from this graph is that under realistic oil field conditions where the sulfide concentrations never exceeds or even approaches 1 M (34 g L^−1^) acetate oxidation is more favorable than sulfide oxidation.

**Figure 5:**
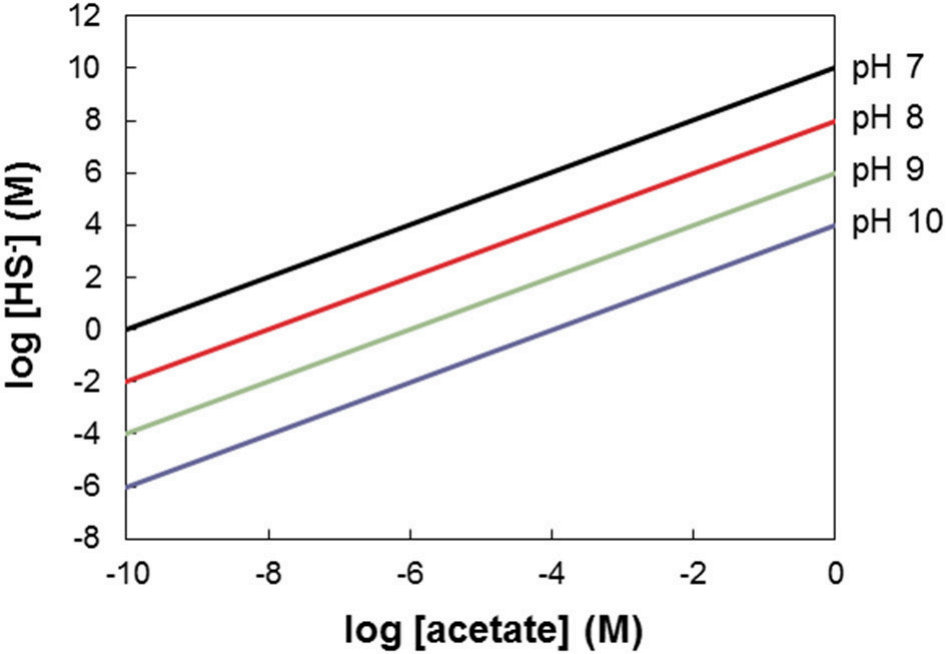
The window of opportunity for acetate driven denitrification (Table 1, R7) vs. sulfide driven denitrification (Table 1, R8), illustrating combinations of sulfide and acetate for which either reaction is most exergonic as function of pH. At pH = 7 sulfide is the most exergonic electron donor for combinations of [HS^−^] and [acetate] plotting above the black line. The black line thus indicates the combinations of acetate and sulfide where the energetics of the two denitrification reactions are equal (cf. Figure 4). At increasing pH values this line is lower on the graph (red, green, and blue lines give the boundaries at pH 8, pH 9, and pH 10, respectively).

Figure 4 assumes conditions of pH 7 and 25°C. The conclusion that acetate is a more favorable electron donor for NRB than sulfide also holds for other pH values. Since protons are reactants in acetate driven sulfate reduction this statement seems trivial for pH < 7 if we employ the stoichiometry CH_3_COO^−^ + ${\text{SO}}_{4}^{2-}$ + 2H^+^ → 2CO_2_ + HS^−^ + 2H_2_O (Table 1, R2) on which Figure 4 is based. However, at pH < 7 H_2_S rather than HS^−^ is the prevalent reduced inorganic species (Stumm and Morgan, 1996). Thus, the stoichiometry to be used is: CH_3_COO^−^ + ${\text{SO}}_{4}^{2-}$ + 3H^+^ → 2CO_2_ + H_2_S + 2H_2_O, which implies that three rather than two moles of protons are consumed per mole of sulfate reduced. At pH > 7 HS^−^ is the prevalent reduced inorganic sulfur species (Stumm and Morgan, 1996). Figure 5 shows the combinations of acetate and HS^−^ concentrations at which the acetate driven sulfate reduction to sulfide is energy neutral (Δ*G* = 0) at higher pH. This reinforces the above conclusion that under *in situ* conditions in oil reservoirs and oil production waters at 25°C acetate oxidation is more favorable than sulfide oxidation.

### Organic electron donors other than acetate

The above conclusion that under real world conditions at 25°C acetate oxidation is more favorable than sulfide oxidation can be generalized to other organic compounds. Figure 1 shows this for a variety of different organic electron donors typically encountered in oil field produced waters (e.g., Barth, 1991; Utvik, 1999), including acetate, propionate, toluene, hexadecane, and naphthalene. Expressed per two electrons transferred the change in standard Gibbs free energy for sulfate reduction to sulfide, for all electron donors, is essentially identical to the Δ*G*°′ values calculated for acetate driven sulfate reduction.

### Incomplete sulfide oxidation

**Figure 6:**
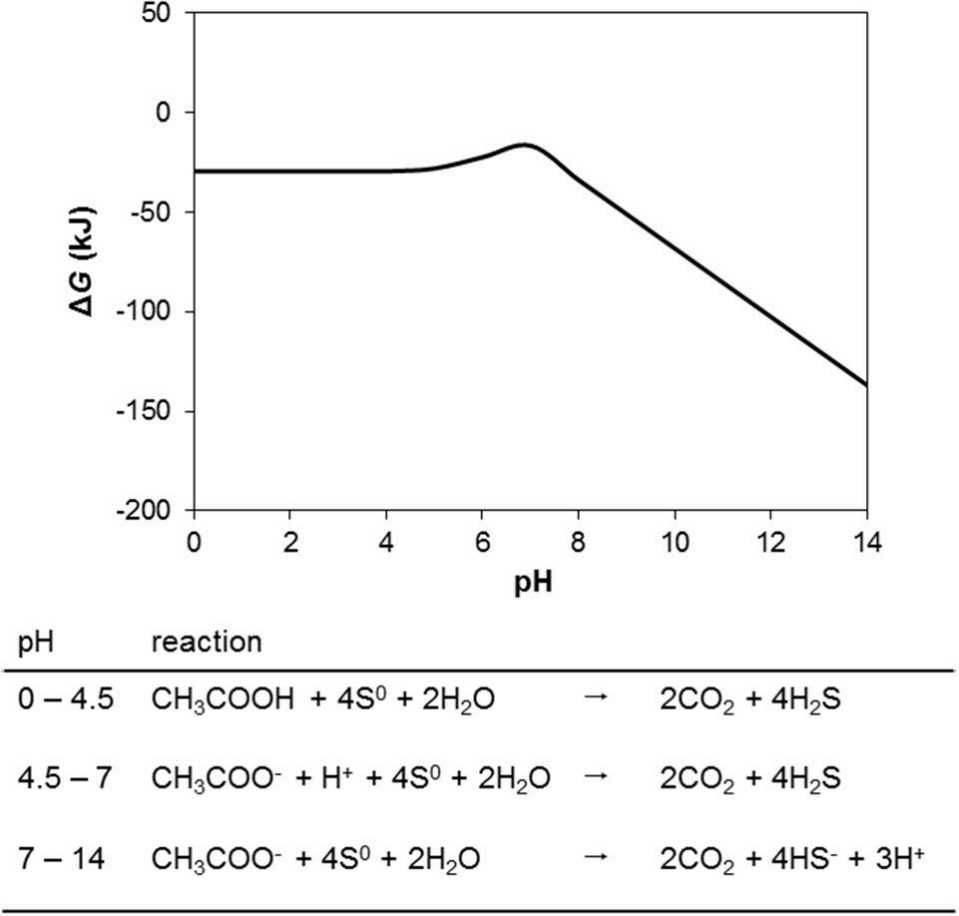
The effect of pH on the change in Gibbs free energy (Δ*G*) of S^0^ driven acetate oxidation under otherwise standard conditions.

The above analysis indicates that under environmentally realistic conditions in oil fields oxidation of organics is always more thermodynamically favorable than sulfide oxidation to sulfate. However, for sulfide oxidation to elemental sulfur the picture changes. Consequences of elemental sulfur being present in the context of souring control are important to consider given that S^0^ and other intermediate sulfur compounds may play an aggressive role in oil field corrosion (Nemati et al., 2001; Hubert et al., 2005; Drønen et al., 2014), a phenomenon causing some operators to critically evaluate nitrate injection technology as a souring control mitigation option. Considering the single reaction “tool” presented above, the change in Gibbs free energy for the reaction CH_3_COO^−^ + 4S^0^ + 2H_2_O ⇆ 2CO_2_ + 4HS^−^ + 3H^+^ is −16.5 kJ/mol under biological standard conditions at pH 7 (Table 1, R10). Low acetate concentrations will drive this reaction to the left (i.e., make sulfide oxidation to S^0^ more favorable than acetate oxidation). At pH 7 the acetate concentration would need to be low enough for the critical ratio between acetate and sulfide would be about 1:1,000 (on a molar basis). Due to the speciation change of H_2_S and HS^−^ at pH 7, low pH has less of an effect on this ratio than would be intuitively expected from the above equation where three moles of H^+^, and four moles of HS^−^, are produced per mole of acetate oxidized. In the range between pH = 4.5 and pH = 7 (the respective pK values for acetate and HS^−^) a lower pH slightly decreases the Δ*G* for the S^0^ driven acetate oxidation (Figure 6).

### Effect of temperature

**Figure 7:**
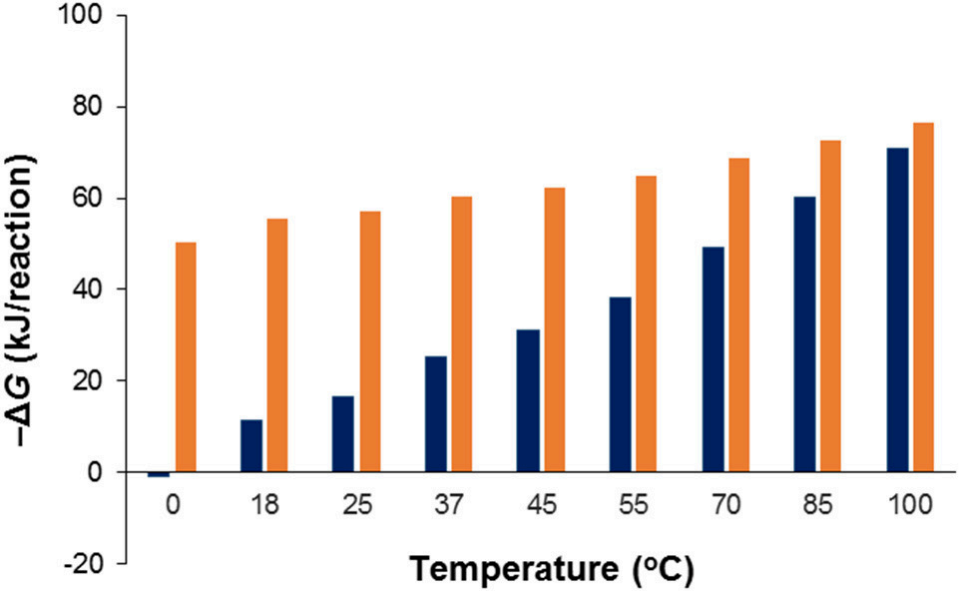
Effect of temperature on the change in Gibbs free energy (Δ*G*) for the acetate driven reduction of ${\text{SO}}_{4}^{2-}$ (Table 1, R1; red bars) and S^0^ (Table 1, R10; blue bars) to sulfide under otherwise standard conditions at pH 7. Note that at low temperature one of the reverse reactions, viz. the oxidation of sulfide to elemental sulfur coupled to CO_2_-based formation of acetate is slightly exergonic.

Acetate oxidation is more favorable than sulfide oxidation to sulfate in the temperature range between 2 and 100°C, and becomes less favorable with decreasing temperatures (Figure 7). For sulfide oxidation to elemental sulfur (S^0^) the effect of temperature is larger and more profound. As illustrated in Figure 7, the energetic advantage of acetate oxidation over sulfide oxidation to S^0^ strongly decreases when temperature decreases, to the extent that at low temperature (2°C) the energetic advantage of acetate disappears. The scenarios depicted for the lower temperatures in Figure 7 may be relevant at topsides oil field facilities in high latitude offshore environments such as the North Sea or farther north in the Barents Sea or the Arctic.

### Denitrification vs. dissimilatory nitrate reduction to ammonia

Denitrification of nitrate to N_2_ is not the only potential nitrate conversion pathway catalyzed by nitrate-reducing microbial communities. Dissimilatory nitrate reduction to ammonia (DNRA) can also be envisaged (Kraft et al., 2014; van den Berg et al., 2015). While denitrifying NRB in oil fields are well known, such as organotrophic *Thauera* and *Pseudomonas* spp. (Agrawal et al., 2012; Fida et al., 2016) and lithotrophic *Sulfurimonas* spp. (e.g., strain CVO; Gevertz et al., 2000), some oil field O-NRB and SO-NRB (e.g., facultatively chemolithotrophic *Sulfurospirillum* spp.) have been shown to reduce nitrate to ammonia in pure culture (Hubert and Voordouw, 2007). Oil fields harboring organisms that reduce nitrate to ammonia are potentially amenable to souring control being achieved with addition of less nitrate, as DNRA is an 8 mol electron transfer reaction per mole of nitrate while denitrification of nitrate to N_2_ is a 5 mol electron transfer reaction per mole of nitrate (Table 1, R5 vs. R3). The lower cost to companies of using less nitrate may be attractive to operators, but this would depend on a knowledge of the ecophysiology of the NRB community present in a given oil production system, e.g., NRB metabolism and whether nitrate gets converted to fully reduced end products (Reinsel et al., 1996; Fida et al., 2016; Okpala et al., 2017).

**Figure 8:**
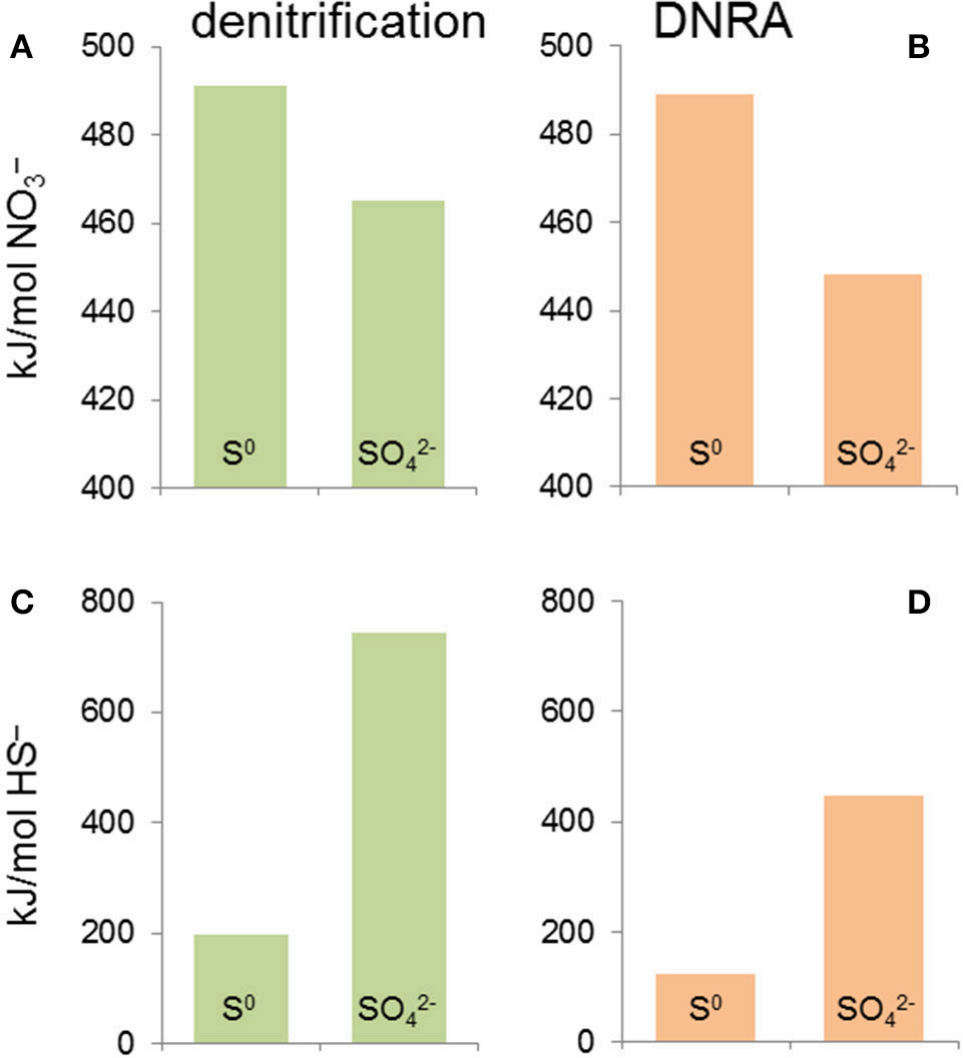
Change in Gibbs free energy values (Δ*G*°′) for sulfide-based nitrate reduction to N_2_ (denitrification) **(A,C)** and to ammonia (DNRA) **(B,D)**. **(A,B)** show the Δ*G*°′ expressed per mole of nitrate reduced, whereas **(C,D)** show the Δ*G*°′ per mole of sulfide oxidized. This predicts that when nitrate is limiting, incomplete oxidation of sulfide to S^0^ is likely to prevail, whereas when sulfide is in short supply (i.e., application of a higher nitrate dose) complete oxidation to sulfate is expected to prevail. These predictions have important implications for managing corrosion risk in nitrate injection scenarios.

There are two metrics that need to be assessed when evaluating the energetics of nitrate based sulfide oxidation: the Δ*G* per mole of nitrate used and the Δ*G* per mole of sulfide oxidized. Figure 8 illustrates that per mole of nitrate used incomplete sulfide oxidation to S^0^ with nitrate as the electron acceptor yields slightly more energy than complete oxidation to sulfate, independent of whether nitrate is reduced to N_2_ or ammonia (Table 1, reactions R8, R9, R11, and R12). When expressed in kJ/mol sulfide oxidized, the differences between the various scenarios are larger. Denitrification yields more energy than DNRA and complete oxidation to sulfate yields more energy than incomplete oxidation to S^0^. When nitrate is limiting, incomplete oxidation of sulfide to S^0^ is likely to prevail, whereas if nitrate is in excess (e.g., a high nitrate dose applied by operators) then complete oxidation to sulfate is expected to prevail.

### Perchlorate or chlorate based souring control interventions

Perchlorate and chlorate have recently been proposed as alternative souring control interventions (Liebensteiner et al., 2014). These alternatives are posited to work via the same mechanisms as nitrate based control interventions: (bio)competitive exclusion and sulfide oxidation. If that assumption is valid, the same analysis as performed here for nitrate based interventions also applies to (per)chlorate based interventions. Perchlorate and chlorate are excellent electron acceptors, with redox potentials at least as favorable as nitrate (Liebensteiner et al., 2014). Therefore, the reasoning outlined in Figure 3 also applies to (per)chlorate: acetate based effects [(bio)competitive exclusion] vs. sulfide oxidation based effects can be rationalized by evaluating the energetics of the reactions CH_3_COO^−^ + ${\text{SO}}_{4}^{2-}$ + 2H^+^ → 2CO_2_ + HS^−^ + 2H_2_O (Table 1, R2) for compete sulfide oxidation when the electron acceptor (nitrate or (per)chlorate) is in large supply, and CH_3_COO^−^ + 4S^0^ + 2H_2_O → 2CO_2_ + 4HS^−^ + 3H^+^ (Table 1, R10) when the electron acceptor is limiting.

### Implications for souring control and corrosion risk

These findings have implications for reservoir souring management and mitigation strategies. For example, there is an abundance of available electron donor in the reservoir, whereas topsides oil/water separation on surface platforms takes place in tanks where nitrate and sulfide may be present in the water but concentrations of acetate (and other organics) may be lower. In this latter context, sulfide oxidation could be most problematic (i) because it is more likely to be expected thermodynamically, and (ii) because of the potential for soNRB mediated corrosion in these topsides settings.

### H_2_ as source of reducing equivalents

**Figure 9:**
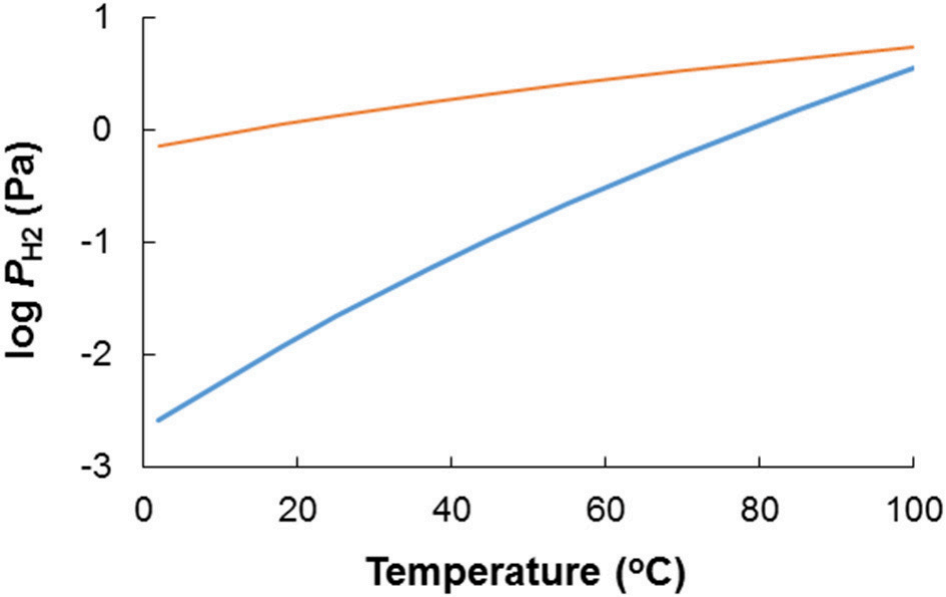
The effect of temperature on the H_2_ “threshold” concentration below which H_2_ is no longer a more favorable electron donor for nitrate reduction than sulfide. The blue line depicts the case where HS^−^ is oxidized to ${\text{SO}}_{4}^{2}$ (Table 1, R2), while the orange line depicts the case where HS^−^ is oxidized to S^0^ (Table 1, R10).

Organics and reduced sulfur compounds are not the only potential electron donors in oil field systems. Hydrogen (H_2_) can also be envisaged as a by-product of anaerobic fermentative metabolism of crude oil compounds (Head et al., 2003), and is an excellent electron donor for both SRB and NRB. The (bio)competitive exclusion of H_2_-oxidizing SRB by H_2_-oxidizing NRB should therefore be considered. A thermodynamic evaluation of H_2_ vs. reduced sulfur compounds as electron donors for NRB in a souring control context follows the same line of reasoning as sketched above for acetate: whether H_2_ or reduced sulfur compounds are the most favorable electron donor can be evaluated based on the change in Gibbs free energy for the reaction 4H_2_ + ${\text{SO}}_{4}^{2-}$ + H^+^ → HS^−^ + 4H_2_O (Table 1, R1). Under otherwise standard conditions at pH 7 H_2_ based sulfate reduction is exergonic (Δ*G*^0^′ = −152 kJ/mol sulfate): The equilibrium for the reaction 4H_2_ + ${\text{SO}}_{4}^{2-}$ + H^+^ ⇆ HS^−^ + 4H_2_O (Table 1, R1) is to the right, which implies that H_2_ is energetically a more favorable electron donor than sulfide, and that hydrogenotrophic NRB would out-compete sulfide oxidizing NRB in a souring control setting. At equimolar concentrations of sulfate and sulfide the threshold H_2_ partial pressure below which sulfide is the most favorable electron acceptor is 0.02 Pa (Figure 9). In reality (i.e., in oil fields) the molar sulfide concentration will be orders of magnitude lower than the sulfate concentration, which further strengthens this assertion. Thus, H_2_ is an energetically more favorable electron acceptor than sulfide when sulfate is the oxidation product. This conclusion is slightly affected by temperature. The *P*_H2_ below which sulfide oxidation is more favorable than H_2_ oxidation decreases from 1.5 Pa at 85°C to an exceedingly low value of 0.003 Pa at 2°C. The sulfide oxidation product has a significant effect on the favorability of H_2_ vs. sulfide as reductant though. The *P*_H2_ below which sulfide oxidation to elemental sulfur is more favorable than H_2_ oxidation ranges from 0.7 Pa at 2°C to 3.5 Pa at 85°C. There are very few data in the literature on hydrogen gas concentrations in petroleum reservoirs (Dolfing et al., 2008). In methanogenic environments H_2_ levels are typically 1–2 Pa, vs. ~0.2 Pa under sulfate reducing conditions (Lovley and Goodwin, 1988; Hoehler et al., 1999). Thus, whether H_2_ or sulfide is the most favorable electron donor for NRB in oil fields will be strongly affected by the local conditions, and cannot be as easily predicted from first principles, compared to the situation for organic compounds such as acetate. It is therefore important to understand the anaerobic microbial communities and metabolic networks whereby oil-derived electron donors promote souring (sulfate reduction) in the subsurface, and the extent to which addition of nitrate as thermodynamically more favorable electron acceptor will divert these substrates to nitrate reduction pathways to control souring.

### Caveats

Our analyses are based on the assumption that all the reactions under consideration can be performed by the organisms present, i.e., that the O-NRB and SO-NRB in question are always found in oil field microbial communities, and would become activated under the chemical and environmental conditions described and assumed in the above scenarios. Obviously this is not necessarily the case in all instances or environments; it may well be that some of the organisms are absent, or are present but unable to be active, or to catalyse the reactions assumed above (e.g., complete reduction of nitrate to end products), or require a certain lag time to build up an effective population. Ongoing studies of subsurface microbial diversity and potential are continually assessing these parameters (Fida et al., 2016; Li et al., 2017; Okpala et al., 2017; Suri et al., 2017; Vigneron et al., 2017). Another caveat is that organisms may act as mixotrophs, for example use sulfide and nitrate and organics and nitrate simultaneously (Hubert and Voordouw, 2007). Another caveat is that other factors like kinetics may trump thermodynamic paradigms for predicting metabolic responses in mixed microbial systems (Chen et al., 2017). Hence thermodynamic calculations should be treated with caution when used as a predictive tool by operators in considering souring mitigation strategies and options. Furthermore, we are aware that introducing various partial pressures or concentrations of CO_2_ can have a profound influence on the reaction pathway in anaerobic ecosystems (cf. Mayumi et al., 2013). While those constraints are needed to make definite statements on the thermodynamics in the systems, we have worked with CO_2_ at atmospheric pressure, as this will provide a baseline for comparisons of reaction energetics.

## Conclusion

The aim of this work is to provide a thermodynamic framework to evaluate the energetics of the various pathways potentially involved in nitrate-facilitated oil reservoir souring control. Nitrate injection technology is based on the textbook premise that nitrate is an energetically more favorable electron acceptor than sulfate. However, this does not necessarily imply that nitrate-facilitated souring control works via direct competition between NRB and SRB for organic electrons donors. An alternative mechanism in which the SRB use the reducing equivalents in the organics to reduce sulfate to sulfide followed by re-oxidation of the produced sulfide by NRB is also relevant, and has been interpreted in field settings following nitrate injection (Telang et al., 1997). The key observation put forward in the present work is that these alternative nitrate reduction mechanisms can be assessed by evaluating the thermodynamics of the difference between these two reactions. Our analysis indicates that, with acetate as a model organic electron donor (Figure 3) sulfate reduction to sulfide is always more energetically favorable than the reverse reaction under realistic oil field conditions. This approach thus predicts that acetate would be a more favorable electron donor than sulfide, e.g., for NRB and hence in a nitrate-based souring control context. Thus, to answer the question phrased in the introduction: nitrate-sulfate competition seems a more likely souring control mechanism than nitrate driven sulfide oxidation, with nitrate reduction fuelled by acetate and other organic compounds as the pathway of choice. However, sulfide is not necessarily oxidized fully to sulfate under all conditions. Incomplete oxidation of sulfide to elemental sulfur by SO-NRB can also be envisaged, especially under conditions where nitrate is limiting. This changes the essential thermodynamics of the reactions under consideration significantly; under nitrate limited conditions, where sulfide is oxidized to sulfur, this reaction can be the energetically more favorable outcome; this is especially true when acetate concentrations are low, and or at low temperatures—conditions that are relevant for oil field topsides facilities where corrosion, possibly accelerated by elemental sulfur, is a major concern. The work presented here offers a simple thermodynamic approach to rationalize the most likely potential outcomes of souring control interventions, and enable predictions around nitrate-based souring control management by oil producers and operators. An additional crucial implication of the considerations made in our study is that half-hearted measures with limited nitrate supplements can be counterproductive as they may be contribute to the formation of S^0^.

## Author contributions

JD: conceived the idea; JD and CH: designed the project, conducted the analyses, and wrote the manuscript.

### Conflict of interest statement

The authors declare that the research was conducted in the absence of any commercial or financial relationships that could be construed as a potential conflict of interest.
